# The Inferior Colliculus in Alcoholism and Beyond

**DOI:** 10.3389/fnsys.2020.606345

**Published:** 2020-12-11

**Authors:** Tanuja Bordia, Natalie M. Zahr

**Affiliations:** ^1^Neuroscience Program, SRI International, Menlo Park, CA, United States; ^2^Department of Psychiatry and Behavioral Sciences, Stanford University School of Medicine, Stanford, CA, United States

**Keywords:** Wernicke’s encephalopathy, Korsakoff’s syndrome, metabolism, energy, ethanol

## Abstract

Post-mortem neuropathological and *in vivo* neuroimaging methods have demonstrated the vulnerability of the inferior colliculus to the sequelae of thiamine deficiency as occurs in Wernicke-Korsakoff Syndrome (WKS). A rich literature in animal models ranging from mice to monkeys—including our neuroimaging studies in rats—has shown involvement of the inferior colliculi in the neural response to thiamine depletion, frequently accomplished with pyrithiamine, an inhibitor of thiamine metabolism. In uncomplicated alcoholism (i.e., absent diagnosable neurological concomitants), the literature citing involvement of the inferior colliculus is scarce, has nearly all been accomplished in preclinical models, and is predominately discussed in the context of ethanol withdrawal. Our recent work using novel, voxel-based analysis of structural Magnetic Resonance Imaging (MRI) has demonstrated significant, persistent shrinkage of the inferior colliculus using acute and chronic ethanol exposure paradigms in two strains of rats. We speculate that these consistent findings should be considered from the perspective of the inferior colliculi having a relatively high CNS metabolic rate. As such, they are especially vulnerable to hypoxic injury and may be provide a common anatomical link among a variety of disparate insults. An argument will be made that the inferior colliculi have functions, possibly related to auditory gating, necessary for awareness of the external environment. Multimodal imaging including diffusion methods to provide more accurate *in vivo* visualization and quantification of the inferior colliculi may clarify the roles of brain stem nuclei such as the inferior colliculi in alcoholism and other neuropathologies marked by altered metabolism.

## Introduction

Alcohol Use Disorder (AUD, 12-month US prevalence 13.9%; lifetime US prevalence 29.1%) is a common mental-health issue and a leading global cause of illness and mortality (Grant et al., 2015). Neuroscience research has aimed at elucidating the brain structures and functions that are impaired by chronic alcohol consumption as well as those that are amenable to repair with sustained abstinence (Salib et al., 2018; Centanni et al., 2019; Fritz et al., 2019; Koob and Colrain, 2020). While it is recognized that alcoholism involves disrupted thalamocortical and pontocerebellar circuitry (Sullivan and Pfefferbaum, 2005), the purpose of this review is to draw attention to a brainstem structure (i.e., inferior colliculus) that has long been recognized as involved in certain aspects of alcohol exposure and withdrawal, but which nevertheless warrants greater attention in the field. The midbrain tectum (i.e., “roof” of the midbrain, also known as tectal plate, and includes the corpora quadrigemina comprising the superior and inferior colliculi) is relatively homologous in all vertebrates but has demonstrated an evolutionary trend toward increased complexity (Maximino, 2008). In considering the role of the inferior colliculus in alcoholism, an overview of the its involvement in the neurological disorder Wernicke-Korsakoff Syndrome and relevant preclinical animal models is first presented. Other disorders are reviewed to highlight a potentially common mechanism of inferior colliculus vulnerability to disruption of normal energy utilization. The review concludes with a hypothesis that alcoholism is associated with reduced synchronization of thalamocortical and pontocerebellar pathways due to inferior colliculi pathology.

## Thiamine Deficiency: Wernicke-Korsakoff Syndrome (WKS)

The phosphate derivatives of the essential, water soluble nutrient—thiamine (vitamin B1) —are required for a number of aerobic processes. The citric acid cycle, for instance, responsible for the regulation of carbohydrate, lipid, and amino acid metabolism, requires thiamine pyrophosphate (TPP, also known as thiamine diphosphate, TDP); TPP is furthermore involved in production of neurotransmitters such as glutamate and GABA (Thompson and McGeer, 1985; Dodd et al., 1996). Thiamine has a half-life of 18 days; 2–3 weeks reserves in the human body are thus readily exhausted, particularly when metabolic demands exceed intake. Although rare in Western countries (Center for Disease Control and Prevention, 2012; National Institutes of Health Office of Dietary Supplements, 2017), thiamine deficiency expressed as Wernicke’s Encephalopathy (WE) may occur in diabetes (Page et al., 2011; Pácal et al., 2014), cancer (Isenberg-Grzeda et al., 2016), hyperemesis gravidarum (Oudman et al., 2019), HIV infection (Kv and Nguyễn, 2013), and in the critically ill (Attaluri et al., 2018); it also occurs following bariatric surgery (Aasheim, 2008; Oudman et al., 2018b) and in individuals with AUD (Thomson et al., 2008; Zahr et al., 2011). Classically, diagnosis of WE required the presence of a clinical triad of oculomotor abnormalities (nystagmus or ophthalmoplegia), cerebellar dysfunction (loss of equilibrium, incoordination of gait, trunk ataxia, dysdiadokokinesia and, rarely, limb ataxia or dysarthria), and altered mental state (mental sluggishness, apathy, impaired awareness, inability to concentrate, confusion or agitation, hallucinations, behavioral disturbances, or coma) (Harper et al., 1986; Victor et al., 1989; Sechi and Serra, 2007).

Practically, WE symptoms are subtle and nonspecific and include loss of appetite, headaches, fatigue, concentration difficulties, irritability, and abdominal discomfort (Jung et al., 2012). Indeed, a retrospective analysis of clinical symptoms of patients diagnosed with WE at autopsy revealed that only 20% presented with the full triad of clinical features and 30% exhibited only cognitive impairment (Harper et al., 1986). Operational criteria to improve clinical diagnosis found that the presence of just two of four signs—dietary deficiency, ocular motor abnormality, cerebellar dysfunction, and either altered mental state or mild memory impairment—was sufficient to identify patients at risk of WE (Caine et al., 1997). The prognosis of WE critically depends on the time of onset (Harper et al., 1986) and dose (Oudman et al., 2019) of thiamine supplementation. If left untreated (as is too frequently the case, e.g., Isenberg-Grzeda et al., 2012), WE can lead to Korsakoff’s syndrome (KS), a severe, typically permanent, neurological disorder characterized by anterograde amnesia (Butters, 1981). The term “Wernicke-Korsakoff syndrome (WKS)” is used to refer to the presence of both WE and KS, due to the close relationship between the disorders (Feinberg, 1980; Butters, 1985).

Archetypal neuropathological reports of the thiamine deficient, WKS brain describe bilateral, symmetric lesions affecting periventricular areas, midbrain tectum, and hypothalamus (Victor et al., 1971). Although damage to the mammillary bodies is frequently present, it is not a necessary concomitant of WE (Victor et al., 1989). More contemporary post-mortem studies of WKS brains also report neuronal loss in thalamus (anterior principal and mediodorsal nuclei) (Halliday et al., 1994; Harding et al., 2000), basal forebrain (Halliday et al., 1994; Cullen and Halliday, 1995), and cerebellar vermis (Phillips et al., 1987). In acute WE, *in vivo* Magnetic Resonance Imaging (MRI) studies recapitulate post-mortem findings in reporting bilateral, symmetrical signal intensity changes (hyperintense on T2-weighted and hypointense on T1-weighted images) representing edemic foci in medial thalamus, mamillary bodies, tectal plate (superior and inferior colliculi), periaqueductal gray, and tissue surrounding the third ventricle (Lenz et al., 2002; Hegde et al., 2011; Ha et al., 2012). Regions sporadically noted by imaging studies as affected in acute WE include the caudate, red nucleus, olivary bodies, cranial nerve nuclei, corpus callosum, cerebellum, pons, and cerebral cortices (Murata et al., 2001; Zuccoli and Motti, 2008; Zuccoli and Pipitone, 2009; Liou et al., 2012). Following the resolution of edema and inflammation of acute WE, quantitative neuroimaging studies indicate volume deficits in affected brain regions (Sullivan and Pfefferbaum, 2009) including mammillary bodies, other hypothalamic nuclei, hippocampus, cholinergic forebrain, pons, and cerebellum (Sheedy et al., 1999; Sullivan et al., 1999; Sullivan and Pfefferbaum, 2009).

Damage to the inferior colliculus, as occurs in WKS, implicates effects on hearing and the vestibular system. Case reports demonstrate impaired vestibulo-ocular reflexes (Probst, 1983; Kattah et al., 2013, 2018) and occasionally hearing loss (Buscaglia and Faris, 2005; Flabeau et al., 2008; Jethava and Dasanu, 2012; Walker et al., 2014) in WE. A retrospective study of 26 WE patients (14 female) showed altered signal intensities in midbrain tectum (superior and inferior colliculi) in 38% of cases (Zuccoli and Pipitone, 2009). The inferior colliculi may be especially vulnerable to rapid thiamine depletion, as may occur in the sequalae of parenteral hyperalimentation (Vortmeyer et al., 1992; Kishimoto et al., 2012). MRI studies occasionally report overt pathology involving the quadrigeminal plate such as the presence of cancer or cysts (e.g., Mancuso et al., 1988; Weindling et al., 1988; Herrmann et al., 1992; Fischer et al., 1994; Ono et al., 1998), but quantitative volumetric measures of the quadrigeminal plate are scarce (cf., Aiba et al., 1997; Angeles Fernandez-Gil et al., 2010; Columbano et al., 2010). To our knowledge, MRI-based inferior colliculi volume in WKS has not been reported.

## Thiamine Deficiency: Animal Models

Animal models permit the study of underlying mechanisms, enabling researchers to better interpret findings from human studies. Two experimental approaches are used to model WE in animals. The slower approach uses a thiamine-deficient diet (i.e., feeding with a thiamine-deficient chow), which can take 3–4 weeks to produce symptoms in rodents (Nakagawasai et al., 2001; Nakagawasai, 2005). Behavioral symptoms can be achieved in just 2 weeks using a combination of a thiamine-deficient chow and intraperitoneal (i.p.) administration of a thiamine pyrophosphokinase inhibitor such as pyrithiamine (Hakim and Pappius, 1981; Zhang et al., 1995; Pfefferbaum et al., 2007; Hazell and Butterworth, 2009). Both models result in symptoms that mimic those observed in human WE, including weight loss, ataxia, seizures, and memory impairment (Pitkin and Savage, 2001; Savage et al., 2012).

In rats, histopathological findings indicate significant neuronal loss and gliosis in the thalamus, hypothalamus, midbrain (vestibular nuclei, inferior olives), and collicular plate (Troncoso et al., 1981; Vortmeyer and Colmant, 1988); the basal forebrain, white matter, and cortical regions are also sometimes affected (Langlais et al., 1996; Langlais and Zhang, 1997). Similar findings showing damage common to periaqueductal gray, mammillary bodies, and inferior colliculi were reported in cats; mediodorsal thalamic damage was reported in fewer than half of animals (Irle and Markowitsch, 1982). Bouts of thiamine deficiency (1, 2, or 4) conducted in groups of three rhesus monkeys showed the inferior colliculi to be among the first affected structures. By contrast, damage to the mammillary bodies and mediodorsal thalamus was not evident even following four bouts of thiamine deficiency (Witt and Goldman-Rakic, 1983). A histological study conducted 6-months following resolution of thiamine deficiency in the rhesus monkey showed persistently significant neuronal loss specific to the inferior colliculi and midbrain cranial nerve nuclei (Cogan et al., 1985). Longitudinal structural MRI findings in thiamine-deficient animals show similar patterns of brain changes including hyperintense signals observed on T2-weighted images in thalamus, hypothalamus, mammillary bodies, hippocampus, and colliculi (Jordan et al., 1998; Pfefferbaum et al., 2007; Dror et al., 2010; Zahr et al., 2014a, 2016b). In cats (Palus et al., 2010; Moon et al., 2013) and dogs (Garosi et al., 2003), hyperintense lesions as a result of thiamine deficiency are also noted in thalamus and colliculi, as well as in cerebellum.

A number of reports using thiamine deficiency models observed that the intensity of neurological symptoms and the extent and location of lesions is complex and can vary greatly among individual animals (Witt, 1985; Read and Harrington, 1986; Mair et al., 1988). Selective vulnerability of thiamine-sensitive regions has been ascribed to their high metabolic demand (Hakim and Pappius, 1981), associated with low energy (ATP and phosphocreatine) (Aikawa et al., 1984), acidosis (reduced pH) (Hakim, 1984; Navarro et al., 2008), reduced carbon dioxide (CO_2_) (Gibson et al., 1989) and elevated nitric oxide (Kruse et al., 2004) production, altered perfusion (Hakim, 1986), compromised blood brain barrier integrity (Phillips and Cragg, 1984; Calingasan et al., 1995; Harata and Iwasaki, 1995; Chen et al., 1997), and gliosis (Leong et al., 1994, 1996). The variously sensitive regions may be vulnerable due to unique underlying mechanisms (e.g., Vortmeyer and Colmant, 1988; Matsushima et al., 1997; Hazell et al., 1998; Meng and Okeda, 2003; Ke and Gibson, 2004; Vemuganti et al., 2006). The edematous nature of inferior colliculus pathology (Watanabe and Kanabe, 1978), for example, may explain why it is detected early in the course of in thiamine deficiency by *in vivo* MRI, which is sensitive to brain injury caused by tissue edema (Jung et al., 2012).

## Alcohol Use Disorder (AUD)

Alcohol Use Disorder (AUD) is a prevalent, complex, dynamic condition with profound CNS effects (Grant et al., 2015; Sullivan and Pfefferbaum, 2019). Chronic alcohol abuse is associated with decreased absorption of thiamine (Hoyumpa, 1980; Lieber, 2003; Martin et al., 2003; Saad et al., 2010; Heirene et al., 2018; Oudman et al., 2018a; Karakonstantis et al., 2020) and impaired hepatic function (Levy et al., 2002; Butterworth, 2009), which may together contribute to subclinical thiamine deficiency. Genetic mutations of the thiamine transporters (SLC19A2/SLC19A3) (Kono et al., 2009) or transketolase enzymes (TKTL1) (Coy et al., 1996, 2005) may further predispose individual alcoholics to thiamine deficiency (Jung et al., 2012). Traditional clinical pathological methods demonstrate only mild cerebral atrophy and lower mean brain weight in cases of uncomplicated (i.e., absent diagnosable neurological complications) alcoholism relative to healthy controls (Harper and Blumbergs, 1982; Halliday et al., 1993).

Quantitative studies, required to characterize the relatively subtle structural abnormalities caused by the direct effects of alcohol, have demonstrated greater mean peri-cerebral spaces in the AUD than in the healthy control brain (Harper et al., 1985). Stereometric studies have suggested that this reduction in brain volume is largely accounted for by the shrinkage of white matter (Harper et al., 1985; de la Monte, 1988; Kril et al., 1997). Cerebellar white matter volume, especially in the vermis, is significantly smaller than in control brains (Phillips et al., 1987), and corpus callosum area is significantly thinned in alcoholics (Harper and Kril, 1988; Tarnowska-Dziduszko et al., 1995). Microscopic studies also reveal ∼25% loss of pyramidal neurons in the superior frontal and frontal association cortices of AUD relative to healthy brains (Harper and Kril, 1994; Kril et al., 1997). Although neuronal loss in the supraoptic and paraventricular nuclei of the hypothalamus correlates with maximum daily alcohol consumption (Harding et al., 1996), pathological studies have not consistently shown a decrease in the number of neurons in cerebellum, basal ganglia (Harper et al., 2003), hippocampus (Kril et al., 1997; Baker et al., 1999), or serotonergic raphe nuclei (Baker et al., 1996).

In general, cross-sectional magnetic resonance imaging (MRI) studies of AUD report volume deficits in cortical gray and white matter and anterior cerebellum (Zahr, 2014). Selective regions of frontal cortex are among the most commonly described volume deficits in alcoholism (Zahr et al., 2017). Large-scale longitudinal MRI studies demonstrate AUD-related volume deficits in frontal, temporal, parietal, cingulate, and insular cortices with evidence for accelerated aging in volumes of precentral and superior frontal cortices (Sullivan et al., 2018). Although less severe, the AUD brain shows volume deficits in regions affected by thiamine-deficiency-associated WKS including mammillary bodies, hippocampus, thalamus, cerebellum, and pons (Sullivan and Pfefferbaum, 2009; Le Berre et al., 2014; Pfefferbaum et al., 2018). These graded effects suggest that AUD individuals carry a history, or “scar,” from subclinical bouts of thiamine deficiency. This hypothesis is supported with reference to neuropsychological performance in studies which categorize AUD individuals by the operationalized criteria for determining history of preclinical WE (Ambrose et al., 2001a,b). Uncomplicated alcoholics meeting no WE criteria performed at normal levels on a large neuropsychological test battery; those meeting one criterion performed at impaired levels on a few of the test composites; those meeting two or more criteria were impaired on all test composites (Pitel et al., 2011; Fama et al., 2019). Thus, although these AUD individuals had no history of clinically diagnosable WE, performance impairment level conforms to the “dose effect” of a WE burden. To our knowledge, only two studies provide evidence for effects of AUD on the tectal plate. A post-mortem neuropathological study comparing alcoholic to control brains (n = 9 in each group) demonstrated higher levels of gangliosides in alcoholics relative to controls in the quadrigeminal plate (Alling and Bostrom, 1980). In a computed tomography (CT) study comparing 327 chronic alcoholics to 419 age-matched controls, the cistern of the quadrigeminal plate was one of six parameters that distinguished alcoholics and controls (Kohlmeyer et al., 1986).

## Animal Models of Alcohol Exposure

Rat models of alcohol dependence, typically using intragastric (e.g., French, 2001), intraperitoneal (e.g., Correa et al., 2009; Fernandez-Lizarbe et al., 2009), or vapor (e.g., Roberts et al., 2000; Vendruscolo and Roberts, 2014) ethanol exposure, have revealed a variety of susceptible brain regions. Markers of degeneration typically highlight effects of ethanol on corticolimbic circuitry (Geisler et al., 1978; Grupp and Perlanski, 1979; Carlen and Corrigall, 1980; Devenport et al., 1981; Roulet et al., 1985; Cadete-Leite et al., 1989; Moghaddam and Bolinao, 1994; Collins et al., 1996, 1998; Nakano et al., 1996; Zou et al., 1996; Corso et al., 1998; Nixon and Crews, 2002; Obernier et al., 2002; Rice et al., 2004; Kelso et al., 2011; McClain et al., 2011; Maynard and Leasure, 2013). Studies determining localized central nervous system (CNS) changes in glucose metabolism—a marker for neuronal activity—show a more widespread signature of ethanol exposure in auditory circuitry (i.e., inferior colliculus, medial geniculate), and structures including thalamus, cerebellum, and pons (Eckardt et al., 1988; Grunwald et al., 1993; Williams-Hemby and Porrino, 1994; Dudek et al., 2015). Animals withdrawing from ethanol show elevated glucose metabolism in the limbic system (i.e., piriform cortex, amygdala, hippocampus), frontal sensorimotor systems, diencephalon (i.e., thalamus, hypothalamus), midbrain (i.e., inferior colliculus, locus coeruleus, median raphe), cerebellum (flocculus, paraflocculus, vermis, white matter), and brainstem (i.e., pons) (Campbell et al., 1982; Eckardt et al., 1986, 1992; Marietta et al., 1986). Similarly, upregulation of immediate early gene (e.g., c-Fos) expression (another marker of recent neuronal activity) during ethanol withdrawal is observed in regions including the olfactory bulbs, cerebral cortex, inferior colliculi, cerebellum, and brain stem (Matsumoto et al., 1993; Wilce et al., 1994; see also, Putzke et al., 1998; Smith et al., 2019).

One of the most consistent, translational findings made with structural MRI in ethanol -exposed rodents is ventricular enlargement, which may be influenced by both timing and exposure method. Rats that achieve binge-like blood alcohol levels via gavage feeding show a larger effect (Zahr et al., 2010b, 2013, 2014b) than rats exposed to ethanol chronically via vapor (Pfefferbaum et al., 2008; Zahr et al., 2020a). The effect on ventricle size is transient: ventricular volume recovers rapidly within one week of abstinence (Zahr et al., 2016a, 2020a,b). A high field strength 7T animal scanner and voxel-based, rather than region-of-interest (ROI) based morphological evaluation demonstrated in animals exposed to binge ethanol exposure reversible ventricular enlargement and thalamic shrinkage but enduring shrinkage of pretectal nuclei, and superior and inferior colliculi (Zhao et al., 2018). In a follow up study, two experiments [binge (4-day) intragastric ethanol in Fisher 344 rats and chronic (1-month) vaporized ethanol in Wistar rats] showed similarly affected brain regions including retrosplenial and cingulate cortices, dorsal hippocampi, central and ventroposterior thalami, superior and inferior colliculi, periaqueductal gray, and corpus callosum: volumes of the colliculi and periaqueductal gray showed persistent deficits with abstinence (Zhao et al., 2020). Although the inferior colliculi, in particular, have been studied as a substrate for ethanol -withdrawal induced seizures (McCown and Breese, 1990, 1993; Chakravarty and Faingold, 1998; Evans et al., 2000; Faingold et al., 2000; N’Gouemo and Morad, 2003, 2014; N’Gouemo et al., 2006, 2015; Akinfiresoye et al., 2016; Newton et al., 2018), this recent study (i.e., Zhao et al., 2020) demonstrates inferior colliculi shrinkage in the response to both acute and chronic ethanol exposure absent seizures and absent detectable thiamine deficiency (cf., Zahr et al., 2010a).

## Other Diseases Involving the Inferior Colliculi

Beyond ethanol, lead (Bini and Bollea, 1947), mercury (Oyanagi et al., 1989; Nagashima, 1997; Kern et al., 2012), and methyl bromide (Goulon et al., 1975) poisoning can result in a WKS-like pattern of brain damage particularly affecting inferior colliculi and visual and auditory functions (Otto and Fox, 1993; Counter et al., 2011).

Lesions in Leigh’s disease—a genetic neurometabolic disorder associated with reduced or absent thiamine triphosphate—are cardinally present in dorsal midbrain (periaqueductal gray, superior and inferior colliculi) among other thiamine-sensitive regions (e.g., thalamus) (Cavanagh and Harding, 1994; Huang et al., 1996; Scalais et al., 2012; Wei et al., 2018) and may be associated with disturbed hearing and vision. Indeed, a recent report describes a woman with a history of bariatric surgery with near total external ophthalmoplegia and hearing loss that resolved with intravenous thiamine treatment (Nyce et al., 2020).

Fetal Alcohol Spectrum Disorder (FASD), which refers to the sequalae of alcohol abuse engaged in during pregnancy and is expressed as attention deficit hyperactivity disorder (ADHD) (Aronson et al., 1997), autistic-like behaviors (Nanson, 1992; Harris et al., 1995), and often exhibiting problems with vision, hearing, speech, and postural stability (Church, 1987; Church and Abel, 1998; Wozniak et al., 2019). Microcephaly is a common feature of FASD, but autopsy studies also report abnormalities of the corpus callosum, brainstem, and cerebellum as well as effects on hippocampus and basal ganglia. Post-mortem findings have been confirmed by *in vivo* MRI studies showing FASD effects on corpus callosum, basal ganglia, diencephalon, and cerebellum (Roebuck et al., 1998). Ethanol-exposed infant rodents (Ikonomidou et al., 2000; Olney et al., 2002; Dikranian et al., 2005) and monkeys (Farber et al., 2010) show brain damage to subcortical structures and cerebellum, with evidence for effects on periaqueductal gray and superior and inferior colliculi (Zajac et al., 1988; Phillips et al., 2000; N’Gouemo and Lovinger, 2012) possibly via mechanisms of impaired mitochondrial function or oxidative stress (Chu et al., 2007).

The complex developmental disorder autism may present with symptoms of oculomotor and auditory dysfunction (Rapin, 1988; Miller et al., 1998). Some studies have shown involvement of the inferior colliculi (e.g., Baldwin et al., 2016) among other brainstem structures such as inferior olives, corpus callosum, and cerebellum (Ritvo et al., 1986; Courchesne, 1997; Piven et al., 1997; Bailey et al., 1998; Kemper and Bauman, 1998) and links to mitochondrial dysfunction and disturbed brain energy metabolism (Lombard, 1998; Chauhan and Chauhan, 2006; Palmieri and Persico, 2010; Shoffner et al., 2010; Chauhan et al., 2011; Anitha et al., 2013; Siddiqui et al., 2016). In animal models, the genetic disorder Fragile X Syndrome likewise shows evidence for inferior colliculus involvement (Gonzalez et al., 2019; Kokash et al., 2019; Nguyen et al., 2020). Asphyxia at birth in humans (Schneider et al., 1975; Leech and Alvord, 1977; Roland et al., 1988) and in monkeys (Windle, 1969; Myers, 1972) results in disproportionate injury to thalamus and brainstem nuclei, particularly the inferior colliculi. In adult animals, the inferior colliculi are among the most frequently and seriously damaged regions in response to transient ischemia (Araki et al., 1990; Siman et al., 2005; Pan et al., 2019).

A 1955 study using radioactive tracers to determine local cerebral glucose utilization in the cat brain revealed greatest consumption in brainstem auditory nuclei, nearly three times more than cerebral white matter and two times more than most cortical regions (Landau et al., 1955–1956). Glucose utilization—in species ranging from mice to monkeys—has since been confirmed to be highest (among evaluated brain regions) in the auditory system, particularly in the inferior colliculi (Sokoloff et al., 1977; Sokoloff, 1981; Kennedy et al., 1982; Bryan, 1986). Capillary density (Gross et al., 1986; Klein et al., 1986; Song et al., 2011), blood volume (Cremer and Seville, 1983), blood flow (Jay et al., 1988), and levels of glucose transport proteins (e.g., GLUT1) (Zeller et al., 1997) are also higher in inferior colliculus than in other regions measured. This high metabolic demand may be due to the involvement of the inferior colliculus in the integration of sensory inputs (Houser et al., 2010). High rates of blood flow and aerobic metabolism (Sokoloff et al., 1977; Zeller et al., 1997) likely increase its vulnerability to toxic and traumatic brain injuries (Kanner and Eisenberg, 1957; Denny-Brown, 1962). Because a variety of syndromes with various pathogenic causes can have WKS-like neuropathology, and because the inferior colliculi are among the most metabolically demanding brain regions, it has been proposed that a common etiological factor may be energy deprivation (Simon, 1999) as would occur in mitochondrial disorders (Lestienne and Bataillé, 1994; Althoff et al., 2010).

## Inferior Colliculus: Structure and Function

**FIGURE 1:**
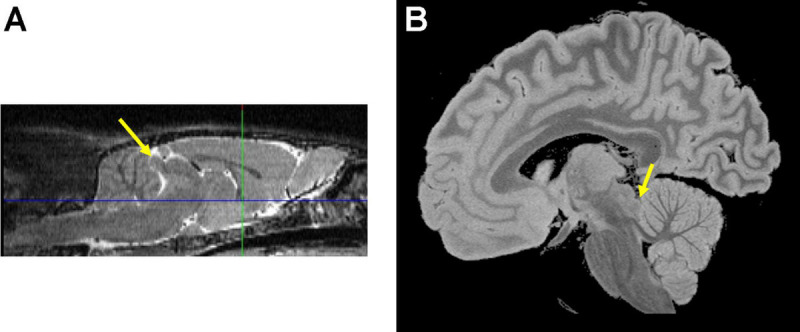
The inferior colliculus (yellow arrows) on sagittal images from a **(A)** wild type Wistar rat and a **(B)** postmortem formalin fixed human brain specimen. Images compliments of Adolf Pfefferbaum, M.D., SRI International.

Located on the dorsal surface of the mesencephalon caudal to the superior colliculus, the inferior colliculus is the largest nucleus of the auditory system. The inferior colliculus varies in size by more than 130-fold among mammals: relative to total brain size, rats have among the largest and humans have the smallest (Glendenning and Masterton, 1998; Figure 1). The inferior colliculus comprises core [central nucleus of the inferior colliculus (CNIC)] and shell (dorsal and ventral nuclei) regions (Oliver and Morest, 1984). Both structures are composed of excitatory glutamatergic and inhibitory GABAergic (∼25%) neurons (Merchán et al., 2005; Ono et al., 2005, 2017; Ito et al., 2011; Schofield and Beebe, 2019). Vascular supply to inferior colliculus is principally via paramedian branches of the basilar artery (Ruchalski and Hathout, 2012; Komune et al., 2015).

The inferior colliculus is one of the earliest structures to become myelinated and functional in the developing human brain. Histological studies have demonstrated that the statoacoustic system begins myelination at the end of the fifth fetal month in the second trimester (Yakovlev and Lecours, 1967; Moore et al., 1995). In mature newborns, the inferior colliculus, superior olivary nucleus, and lateral lemniscus are nearly completely myelinated (Rorke et al., 1968). Early myelination of the inferior colliculi has also been demonstrated *in vivo* by MRI (Curnes et al., 1988; Counsell et al., 2002; Sano et al., 2007).

**FIGURE 2:**
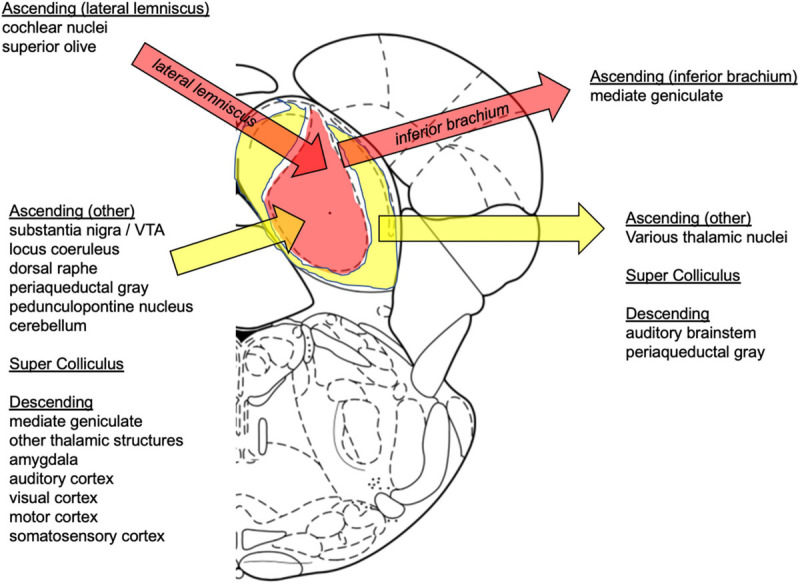
Summary of inferior colliculus afferent and efferent connections. **Afferents:** Ascending (later lemniscus): cochlear nuclei (Klepper and Herbert, 1991; Cant and Benson, 2006), superior olive (Cant and Benson, 2006), lateral lemniscus (Zhang et al., 1998; Saalmann et al., 2006; Kelly et al., 2009; Chen et al., 2018); Ascending (other): substantia nigra/VTA (Takada et al., 1987; Kemel et al., 1988; Yasui et al., 1991; Deniau and Chevalier, 1992; Moriizumi et al., 1992), locus coeruleus (Klepper and Herbert, 1991; Hormigo et al., 2012), dorsal raphe (Hurley and Pollak, 2001; Hurley, 2006; Hurley and Sullivan, 2012), pedunculopontine nucleus (Noftz et al., 2020), periaqueductal gray (Dujardin and Jürgens, 2005), cerebellum (Huffman and Henson, 1990); Super Colliculus (Adams, 1980; Clerici and Coleman, 1987; Stepniewska et al., 2000; Nodal et al., 2005); Descending: medial geniculate (Driscoll and Tadi, 2020), other thalamic structures (Kuwabara and Zook, 2000), amygdala (Hopkins and Holstege, 1978; Marsh et al., 2002), auditory cortex (Games and Winer, 1988); visual, motor, somatosensory cortices (Cooper and Young, 1976). **Efferents:** Ascending (inferior brachium): medial geniculate (Oliver, 1984b; Oliver et al., 1991; Peruzzi et al., 1997; Smith et al., 2006); Ascending (other): other thalamic nuclei (Kudo and Niimi, 1980; Senatorov and Hu, 2002; Smith et al., 2006), auditory cortex (Schofield et al., 2011; Xiong et al., 2015; Schofield and Beebe, 2019; Lesicko et al., 2020); Super Colliculus (Van Buskirk, 1983); Descending: auditory brainstem (Huffman and Henson, 1990), periaqueductal gray (Santos et al., 2003; Reimer et al., 2008).

Afferents to the inferior colliculus are excitatory and inhibitory (González-Hernández et al., 1996; Patel et al., 2017; Figure 2). Brainstem ascending inputs from the cochlear nuclei and superior olives via the lateral lemniscus generally terminate bilaterally in the CNIC (Cant and Benson, 2006, 2007). Additional afferents, which tend to be non-auditory, principally target the shell and arise from other brainstem nuclei (e.g., substantia nigra pars compacta, ventral tegmental area, dorsolateral periaqueductal gray, olivary nuclei, locus coeruleus, dorsal raphe), spinal trigeminal nucleus (somatosensory input), deep layers of the superior colliculus, cerebellum, and target regions such as medial geniculate body, other thalamic structures (posterior limitans, suprapeduncular nucleus, and subparafascicular intralaminar nuclei of the thalamus), and auditory cortex (Adams, 1979; Glendenning and Masterton, 1983; Oliver, 1984a, 1987; Coleman and Clerici, 1987; Winer et al., 1998, 2005; Zhou and Shore, 2006; Loftus et al., 2008; Hurley and Sullivan, 2012; Chen et al., 2018). Indeed, a number of studies show that processing and integration in the inferior colliculi are significantly modulated by a massive descending corticofugal system (Jen et al., 1998; Jen and Zhou, 2003; Popelár et al., 2003; Yan et al., 2005; Zhou and Jen, 2007; Ma and Suga, 2008). Efferents from the inferior colliculus predominately targeting ipsilateral medial geniculate bodies travel through the inferior brachium (Calford and Aitkin, 1983); 40% of projections to the thalamus are GABAergic (Peruzzi et al., 1997). There is also evidence for efferent fibers to lateral lemniscus (Ito and Oliver, 2012; Ruchalski and Hathout, 2012), periaqueductal gray, and superior colliculus (Chen et al., 2018; Goyer et al., 2019).

Positioned to serve as a relay station to analyze, integrate, and route sound signals to higher level brain centers (Casseday et al., 1994; LeBeau et al., 2001; Pan et al., 2004), the inferior colliculus is also involved in multi-modal sensory perceptions such as the vestibulo-ocular reflex (Feng, 1992; Brandao et al., 1993), and startle response (Jordan and Leaton, 1982; Leitner and Cohen, 1985; Moore et al., 1995; Li et al., 1998; Li and Yeomans, 2000; Li and Yue, 2002; Heldt and Falls, 2003; Nobre et al., 2003; Satake et al., 2012). Specifically, converging anatomical and physiological evidence indicates that cells within the inferior colliculus are sensitive to visual, oculomotor, and somatosensory information as well as to signals relating to behavioral context and reward. Ethnologically, it is considered to drive acoustic-motor behaviors including predator escape (Xiong et al., 2015), prey localization (Knudsen and Konishi, 1978; King et al., 1998), and conspecific communication (Jürgens, 2002; Wilczynski and Ryan, 2010; Gruters and Groh, 2012). The inferior colliculus may also serve to enhance perception by decreasing attentional thresholds and increasing alertness (Hermans et al., 2011; LeDoux, 2012); indeed, arousal induced by sound can facilitate attention in a subsequent visual search (Lee et al., 2014; Asutay and Vastfjall, 2017), a behavior likely mediated by the inferior colliculus. The brainstem auditory evoked potential (BAEP) is easily recorded, has an invariant waveform and is stable and robust (Shaw, 1988). Both human (e.g., Kevanishvili, 1980; Zappia et al., 1996) and rodent (e.g., Funai and Funasaka, 1983; Shaw, 1987) studies have ascribed the inferior colliculus as the origin of portions of wave V of the BAEP (Kevanishvili, 1980). A comprehensive discussion of inferior colliculus organization and function is beyond the scope of this review. Instead, the interested reader is referred to Winer and Schreiner (2005) and Malmierca and Young (2015).

## Inferior Colliculi: AUD-Related Neural Circuitry

The remaining portions will discuss the inferior colliculus in the context of alcoholism-related circuitry. Behaviors associated with the use of ethanol include an initial stimulatory effect and a prominent depressant action; alcohol abuse can result in tolerance and physical dependence, which may express as withdrawal comprising symptoms of tremor, hallucinations, motor and autonomic hyperactivity, and seizures. Critically, the inferior colliculus has long been described as involved in ethanol-withdrawal seizures, typically induced by an auditory trigger in animal models (Riaz and Faingold, 1994) and supported by research in genetically epilepsy-prone rats (Faingold et al., 1992; Ribak and Morin, 1995). During ethanol withdrawal, inferior colliculus metabolism is elevated (Eckardt et al., 1986) allowing for enhanced responsivity to acoustic stimuli, thereby providing a basis for greater seizure susceptibility (Chakravarty and Faingold, 1997). This is due, in part, to the fact that GABA-mediated inhibition, which normally limits high intensity firing of inferior colliculus neurons, is less effective during ethanol withdrawal due to downregulation or desensitization of GABA-A receptors and over-activation of NMDA receptors (Faingold et al., 1993).

Independently, work has implicated the periaqueductal gray in responses to ethanol exposure in animal models (Yang et al., 2003; Ezequiel Leite and Nobre, 2012; Li et al., 2013). Both the periaqueductal gray (George et al., 2019) and the inferior colliculus (Nobre et al., 2010) may be involved in stress and anxiety. The quadrigeminal plate and dorsal periaqueductal gray (Bandler et al., 1991; Brandão et al., 1994) and likely amygdala and medial hypothalamus (Coimbra and Brandão, 1997) are the presumed neural substrates of aversion, integration of defensive behaviors, and analgesia (Lowe et al., 2007; Reimer et al., 2008). In particular, the inferior colliculus, which can respond to both rewarding (Nienhuis and Olds, 1978) and aversive (Ruth et al., 1974) stimuli, is thought to integrate information involved in modulating fear-related behaviors (Lamprea et al., 2002) via anatomical and functional connections with the amygdala (Maisonnette et al., 1996). Indeed, neuronal activity in a circuit between the medial prefrontal cortex and dorsal periaqueductal gray (Vander Weele et al., 2018) was recently shown to regulate ethanol drinking: inhibition of this cortico-brainstem pathway promoted compulsive (i.e., aversive-resistant) drinking (Siciliano et al., 2019). These findings, however, are derived from animal models.

Although there has been some support for inferior colliculus involvement in seizures associated with Leigh’s disease (e.g., Wei et al., 2018), direct evidence for their involvement in alcohol withdrawal in human studies is scarce. Instead, BAEP responses to alcohol indirectly suggest involvement of the inferior colliculi (e.g., Squires et al., 1978). BAEPs are particularly impaired in alcoholics with a history of seizures (Touchon et al., 1984; Neiman et al., 1991). Even in healthy human men, however, blood alcohol levels of 70 mg/dL were associated with depression of several components of the BAEP (Pfefferbaum et al., 1979). Ethanol may have greater effect on the BAEPs elicited under inattentive than under attentive conditions (Soveri and Fruhstorfer, 1969) suggesting that the generally observed depressant effect on BAEP is not due solely to the direct pharmacological ethanol but may be mediated or intensified by the general decline in attentiveness accompanying intoxication.

## Perspective

As described, our recent work using novel, voxel-based analysis of structural MRI data demonstrated in three independent studies [two ethanol intoxication models: “binge” (4-day) via intragastric gavage and “chronic” (1-month) via vaporized ethanol; two strains: wild-type Wistar and Fisher 344 rats] significant shrinkage of the inferior colliculi (Zhao et al., 2018, 2020). In Wistar rats exposed to the binge protocol, a single week of abstinence was insufficient for inferior colliculi volume recovery (Zhao et al., 2018). Notably, Wistar rats continued to show transient inferior colliculus volume loss even after three cycles of 1-month vaporized ethanol exposure (Zhao et al., 2020). In both intragastric binge and “chronic” vaporized ethanol models, earlier work failed to demonstrate quantifiable thiamine deficiency (cf., Zahr et al., 2009, 2010a).

Volume loss in response to ethanol in brain regions sensitive to thiamine deficiency may be interpreted in several ways. **(1)** Ethanol-exposed animals could be thiamine-deficient transiently (i.e., missed data collection time point) or below detection levels. This interpretation would also hold for human studies which are even more challenging than those in animals with respect to capturing a clinically relevant time point (i.e., individuals with AUD may experience transient thiamine deficiency that is not captured in a laboratory setting). **(2)** Alternatively, circulating thiamine levels may be adequate, but ethanol may disrupt tissue absorption of thiamine (Abdul-Muneer et al., 2018). **(3)** Finally, high doses of ethanol may disrupt energy metabolism without affecting thiamine utilization. Ethanol, per se, is disruptive to cellular respiration (Blachley et al., 1985; Cunningham and Ivester, 1999). AUD is associated with decreased brain glucose utilization (Volkow et al., 2006; Pawlosky et al., 2010) and increased acetate uptake (Sarkola et al., 2002; Volkow et al., 2013). Evidence suggests that even heavy drinking promotes the use of acetate rather than glucose as a substrate of mitochondrial energy oxidation (cf., Jiang et al., 2013). The ethanol-induced utilization of alternative energy substrates may initially target brain regions with high metabolic demand such as the inferior colliculi.

**FIGURE 3:**
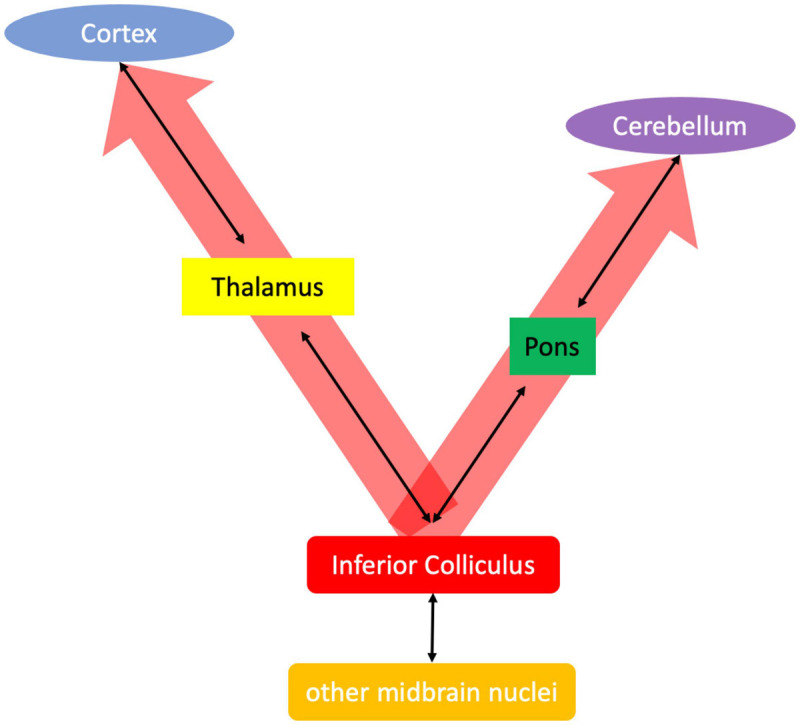
Proposed alerting function of the inferior colliculus accomplished by synchronizing thalamocortical and pontocerebellar systems.

Beyond primary sensation (Sprague et al., 1961), the tectum is a “perceptual facilitating apparatus….a vital facilitator of thalamocortical function…” (so that) just as cortical activity is dependent on thalamic integrity, so both also require the colliculi (Denny-Brown, 1962). It is this more general role—that is, the “alerting” or “attention-focusing” function of the inferior colliculus (cf., Latash, 1990) that is proposed herein to be the initial insult in alcohol addiction. Disruption of thalamocortical circuitry in AUD has been associated with craving (Modell et al., 1990), reduced arousal (Jia et al., 2007; Eckle and Todorovic, 2010), and sleep impairments (Liu et al., 2019), whereas disordered pontocerebellar circuitry has been associated with impaired balance and visuospatial abilities (Sullivan, 2003; Sullivan et al., 2010). Thus, it is proposed that the primary impairment in alcohol exposure is due to a lack of thalamocortical and pontocerebellar synchronization by the inferior colliculi (Figure 3).

A caveat to the proposed hypothesis is that most of the work highlighting ethanol effects on the inferior colliculi has been accomplished in animal models. A phylogenetically older structure such as the midbrain inferior colliculus may be more salient to rodent than human alcohol exposure. Further, the inferior colliculus is sensitive to anesthesia (Kuwada et al., 1989; Szalda and Burkard, 2005; Franken et al., 2008; Huang et al., 2019), adding complexity to interpretations of *in vivo* investigations requiring sedation. Alternatively, as already described, the inferior colliculi are a relatively large structure in rodents, but relatively small in humans making them rather difficult to study. To our knowledge, MRI-based volumetric analysis of the inferior colliculi in AUD has not been accomplished. In conclusion, although the contribution of brainstem nuclei (particularly periaqueductal gray, see George et al., 2019; Siciliano et al., 2019) to aspects of alcohol addiction are now under investigation, it is recommended that greater attention be given to the potential contribution of the inferior colliculus to AUD.

## Author Contributions

NMZ formulated the ideas and concepts expressed herein. TB helped write and endnote portions of the manuscript. Both authors contributed to the article and approved the submitted version.

## Conflict of Interest

The authors declare that the research was conducted in the absence of any commercial or financial relationships that could be construed as a potential conflict of interest.
